# Respirometry‐Based Screening of Marine Natural Products Identifies Leptochelin A as a Novel Modulator of Mitochondrial Function

**DOI:** 10.1155/omcl/9524037

**Published:** 2026-05-26

**Authors:** Howard J. Phang, Adrian M. Arciniega, Jaclyn Bergstorm, Nicole E. Avalon, Evgenia Glukhov, William H. Gerwick, Anthony J. A. Molina

**Affiliations:** ^1^ School of Medicine, University of California San Diego, La Jolla, California, USA, ucsd.edu; ^2^ Skaggs School of Pharmacy and Pharmaceutical Sciences, University of California San Diego, La Jolla, California, USA, ucsd.edu; ^3^ Center for Marine Biotechnology and Biomedicine, Scripps Institution of Oceanography, University of California San Diego, La Jolla, California, USA, ucsd.edu; ^4^ Robert A. Mah Molecular Innovations Center, Department of Pharmaceutical Sciences, School of Pharmacy and Pharmaceutical Sciences, University of California, Irvine, California, USA, berkeley.edu; ^5^ Sam and Rose Stein Institute for Research on Aging, University of California San Diego, 9500 Gilman Drive, 92093, La Jolla, California, USA, ucsd.edu

## Abstract

While mitochondria are recognized as promising therapeutic targets for common pathologies of aging, existing drug discovery platforms fail to capture the adequate physiological and biological contexts necessary to identify translatable, clinically‐relevant leads. The goal of this study was to identify marine natural products that modulate mitochondrial function using a screening pipeline leveraging primary human cells in a cell‐based phenotypic primary screen. Using this approach, we identified leptochelin A, a recently described metallophore, as a candidate hit with strong potency and efficacy towards the inhibition of mitochondrial function. Using high‐resolution respirometry and fluorescence imaging, we validated the mitochondrial‐modulatory (“mito‐modulatory”) effects of leptochelin A and found that it inhibits multiple pathways in the electron transfer system (ETS) while having little effect on mitochondrial mass or superoxide production. It also increases mitochondrial ATP levels, though this may be attributable to a parallel increase in glycolysis. Our findings demonstrate the utility of phenotypic screening using human primary cells to identify novel mitochondrial modulators with translational potential. Leptochelin A’s ability to inhibit mitochondrial function without imposing significant toxicity, coupled with its metal‐chelating properties, make it a unique compound with therapeutic potential for aging and age‐related disorders. Screening strategies focused on mitochondrial respiration can serve as a platform for the advancement of drug discovery within the pharmacology space of human aging.

**Summary**

• We utilized primary human dermal fibroblasts as our screening model, providing robust physiological relevance to human aging.
• Respirometry‐based screening identifies compounds with direct functional effects on mitochondrial biology, validated through high‐resolution respirometry.
• Marine natural products represent an underexplored chemical space with the potential to contribute to new successes in biomedical compound discovery.
• Leptochelin A is a recently described metallophore, posing potential unique advantages in the therapeutic targeting of aging physiology.

## 1. Introduction

Multiple lines of evidence link mitochondrial alterations with aging, suggesting that targeting mitochondrial function may promote healthy aging [1, 2]. This is supported by the growing number of pharmaceuticals in preclinical and clinical development that target mitochondrial pathways to support healthy aging. For example, the antihyperglycemic agent metformin, which inhibits complex‐I and promotes mitochondrial biogenesis, has demonstrated lifespan extension and healthspan improvement in multiple models [3–6]. Similarly, nicotinamide adenine dinucleotide (NAD^+^) precursors like nicotinamide riboside and nicotinamide mononucleotide, which enhance NAD^+^ dependent metabolic processes, have demonstrated efficacy in improving physiological markers of aging in preclinical models (7, 8). NAD^+^ precursors are in multiple phase II and III trials to uncover their applicability to aging and age‐related conditions in humans. The rise of these mitochondrial modulators in the drug development space underscores their considerable potential in the development of healthy aging interventions.

Marine natural products represent an extensive and largely underexplored chemical space with significant therapeutic promise. In contrast to conventional natural products from sources like plants and terrestrial organisms, many marine‐derived molecules have evolved in unique aquatic environments, resulting in extraordinary structural diversity and bioactivities. The adaptations of marine organisms position marine natural products as an underexplored frontier in drug discovery, and only 17 marine‐derived medications are approved by the United States’ Food and Drug Administration for clinical use to date [9]. Given that 70% of the surface of the Earth is comprised of ocean, it is likely that many more marine‐derived bioactive compounds with biomedical applications remain to be discovered [10, 11]. With the advancement of sampling methods, genomic analysis, synthetic chemistry, and chemical computation, marine natural products have the potential to contribute to new successes in biomedical compound discovery [11].

The goal of this study was to identify marine natural product modulators of mitochondrial function. Here, we present a novel screening strategy designed to identify mitochondrial‐modulatory (“mito‐modulatory”) compounds using primary human dermal fibroblasts. Primary human fibroblasts are amenable to large‐scale compound screening and, importantly, serve as a robust model for the study of aging biology in vitro [12, 13]. Traditional screens that utilize protein preparations or animal cell lines strip the experimental environment of relevant human biochemical and physiological contexts. By utilizing primary human cells, we ensure identification of hit compounds with functional impacts on human cell biology. With this approach, we identified leptochelin A as a potent and effective inhibitor of mitochondrial function and further validated its mito‐modulatory effects using high‐resolution respirometry and fluorescence imaging techniques.

## 2. Materials and Methods

### 2.1. Marine Natural Product Library

For the primary screen, we used a library consisting of 127 pure natural product compounds derived from marine organisms from several phyla, such as Cyanobacteriota (blue–green algae), Rhodophyta (red algae), Phaeophyceae (brown algae), Porifera (sponges), and Mollusca (mollusks). This taxonomic breadth was deliberately selected to maximize chemical and bioactive diversity, as the inclusion of under‐explored marine microorganism sources complements other more commonly studied macroorganisms. The list of natural products has been previously described [14]. Compounds were originally directly extracted from source materials and either reisolated or synthesized in sufficient scale for biological testing. Compounds were purified through iterations of liquid chromatography and fully characterized through spectroscopic and spectrometric analysis. Each compound was tested in 0.1, 1, or 10 µg/mL, and each concentration was tested in triplicate.

### 2.2. Cell Culture

For the primary screen and mitochondrial validation, we used primary human dermal fibroblasts collected from the San Diego Nathan Shock Center (SD‐NSC) Clinical Cohort. Dermal fibroblasts retain key biological and phenotypic features of the donor, including age‐related bioenergetic status, making them a useful model for in vitro drug discovery [12, 13, 15–17]. The SD‐NSC Clinical Cohort provides fibroblasts from healthy adults across the human life course, selected through strict inclusion and exclusion criteria to minimize biological variability. Participants from the SD‐NSC have been described in a previous publication [18]. The fibroblasts were grown in DMEM with 15% FBS, 1% nonessential amino acids, and 1% Glutamax. Cells were cultured in T75 flasks until 90% confluence, then lifted using ATV for experimentation.

### 2.3. Respirometry

#### 2.3.1. High‐Throughput Respirometry

We utilized the Agilent Seahorse XFe96 Respirometer (Agilent Inc., Santa Clara, CA) and the Mito Stress Test as our primary screen to identify compounds with effects on cellular respiratory capacity. For our cell model, we chose cells from a 72‐year‐old male SD‐NSC participant to recapitulate an older‐adult bioenergetic phenotype. We plated cells in an XFe96 assay plate at 25,000 cells/well and allowed them to rest at room temperature for 1 h before incubating at 37°C with 5% CO_2_. After 24 h, we replaced culture media with fresh media containing each compound at the concentrations specified above and let the cells incubate with each treatment for another 24 h before experimentation.

We used the Mito Stress Test protocol to measure the oxygen consumption rate of fibroblasts treated with each compound. Assay media consisted of XF DMEM base medium supplemented with 30 mM glucose, 4 mM Glutamax, and 1 mM pyruvate. Prior to experimentation, we washed the cells three times with XF assay medium and added 1 µM rotenone and antimycin A to positive control wells. We allowed each plate to degas for 1 h to allow equilibration of air and medium CO_2_. Our primary outcomes were basal respiration (Basal) and maximal respiration (Max), with spare respiratory capacity (SRC) as a secondary outcome. We recorded Basal using the third measurement after the start of the assay and Max using the highest measurement following BAM15 addition and before rotenone and antimycin A addition. SRC was calculated as the difference between Max and Basal. All values were corrected for nonmitochondrial respiration, calculated as the average of the last three measurements after rotenone and antimycin A addition. We included exploratory outcomes proton leak respiration (Leak) and ATP‐linked respiration (ATP‐L) in Supporting Information. Leak was recorded as the lowest measurement after the addition of oligomycin before BAM15, and ATP‐L was calculated as the difference between Basal and Leak. At the end of each assay, we used 30 μM Hoechst 33,342 to stain cells for counting and normalization.

We also used the Agilent Seahorse XFe96 to identify the IC_50_ of leptochelin A and test the effect of leptochelin A on multiple fibroblast lines. For the identification of leptochelin’s IC_50_, we treated cells with a serial dilution of leptochelin A beginning at 10 μg/mL (11.2 μM) and diluting by half for 10 total concentrations. We then used the Mito Stress Test protocol to measure Basal and Max.

#### 2.3.2. High‐Resolution Respirometry

High‐resolution respirometry provides detailed analysis of multiple entry points of the electron transfer system (ETS). This protocol uses multiple substrates and inhibitors that are sequentially added to measure changes in oxygen flux due to fatty acid oxidation, followed by oxidative phosphorylation. Briefly, we loaded 600,000 cells into a chamber with MiR05 buffer to equilibrate, followed by measurement of routine respiration. Addition of digitonin (permeabilizer), ADP, octanoylcarnitine (fatty acid), malate, cytochrome C, pyruvate, glutamate, succinate, glycerophosphate, FCCP (uncoupler), rotenone, antimycin A, ascorbate, TMPD, and azide allowed us to capture the bioenergetic parameters fatty acid oxidation, complex‐I, complex‐II, maximal OxPHOS, maximal ETS, and complex‐IV mediated respiration. Detailed methodologies have been previously described [19].

### 2.4. Glycolytic Capacity

The Agilent Seahorse XFe96 allows concurrent measurement of glycolytic flux in the form of proton efflux rate, which has been shown to be directly correlated to other measures of glycolytic function such as cellular lactate efflux [20]. We used the glycolytic proton efflux rate (GlycoPER) calculation as described previously [20]. We used GlycoPER both prior to and following the addition of oligomycin to observe basal glycolysis and oligomycin‐stimulated glycolysis.

### 2.5. Fluorescence Imaging

For this study we used commercially available dyes to probe a variety of mitochondrial outcomes. MitoTracker Green (MTG; Thermo Fisher) provides a measure of relative mitochondrial content through its binding to free thiol groups in the mitochondrial matrix [21]. MitoSOX Red (SOX; Thermo Fisher) provides a measure of mitochondrial superoxide level [22]. BioTracker ATP‐Red (ATP‐R; Sigma–Aldrich) provides a measure of mitochondrial ATP level by selectively binding ATP through three distinct steric sites [23]. Tetramethylrhodamine, Ethyl Ester, Perchlorate (TMRE; ThermoFisher) provides a relative measure of mitochondrial membrane potential [23]. Last, Hoechst 33342 provides a quantification of cell number that allows each fluorescence measurement to be normalized to cell count.

Briefly, we plated fibroblasts at 15,000 cells per well on a glass‐bottom 96‐well black plate. After allowing cells to incubate for 24 h, we added 0.84 or 1.68 μM leptochelin A to treatment wells for a 24‐h exposure. Prior to experimentation, we washed out cell media three times and added 10 µM rotenone and antimycin A to positive control wells. After a 20‐min incubation, we washed out the inhibitors three times and added 0.2 µM MTG, 5 µM SOX, 5 µM ATP‐R, or 0.5 µM TMRE to their respective wells. After a 20‐min incubation, we washed out the fluorescent dyes and replaced the media with 100 µL Dulbecco’s phosphate buffered solution (DPBS) for final visualization on a BioTek Cytation 1 (Agilent Inc., Santa Clara, CA). Quantification was performed using Gen5 Version 3.14 software and analyzed using R Version 4.2.2.

### 2.6. Statistical Analyses

We used GraphPad Prism Version 10.2.2 to conduct statistical analyses of respirometric data. For quantification of fluorescence imaging, we used R Version 4.2.2 to generate mixed effects models and conduct statistical comparisons.

## 3. Results

### 3.1. Screening Marine Natural Products for Mitochondrial Modulation in Primary Human Dermal Fibroblasts

**Figure 1:**
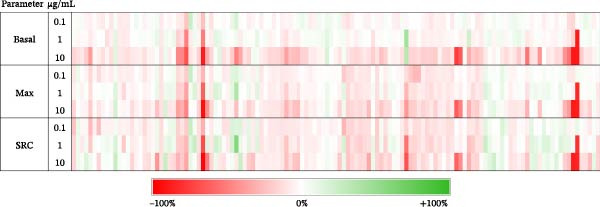
Heatmap of primary screening results. Marine natural products were screened at 0.1, 1, and 10 μg/mL and the percent change in Basal, Max, and SRC were recorded. Red indicates a negative change while green indicates a positive change. SRC, spare respiratory capacity.

For the primary screen, we used the Seahorse XFe96 and Mito Stress Test to identify mito‐modulatory compounds in primary human dermal fibroblasts derived from a 72‐year‐old male donor. We screened 127 pure compounds at 0.1, 1, and 10 μg/mL from the Gerwick Pure Compounds Library using Basal and Max as primary outcomes and SRC as a secondary outcome. Overall, we identified 25 compounds that inhibited basal or maximal respiration, based on a ≥ 20% change from vehicle control. A list of those compounds and their respective percent change in Basal and Max are reported in Table S1. We also identified two compounds that stimulated basal or maximal respiration using the same threshold. Figure 1 shows a heatmap of screening results. Figure S1 shows a heatmap of exploratory outcomes Leak and ATP‐L.

**Figure 2:**
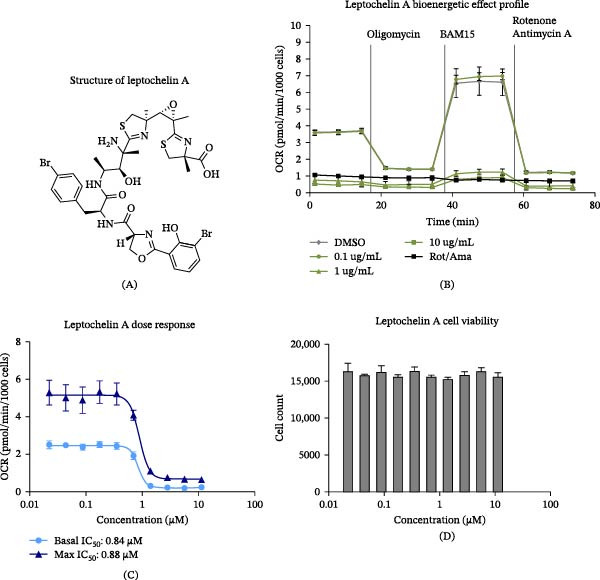
Characteristics of leptochelin A from early screening. (A) Structure of leptochelin A. (B) The bioenergetic effect of leptochelin A determined from the primary screen. (C) Dose response curve demonstrating the IC_50_ of Basal and Max following 24 h treatment with leptochelin A (*n* = 2). (D) Corresponding cell viability data from cells used in the identification of IC_50_ (*n* = 2). DMSO, dimethyl sulfoxide; OCR, oxygen consumption rate.

From the pool of identified compounds, we chose to further characterize and validate leptochelin A (Figure 2A) for its high potency and efficacy. As represented in Figure 2B, 1 and 10 μg/mL of leptochelin A inhibited basal respiration more effectively than the rotenone and antimycin A positive controls. A dose response revealed the IC_50_ to be 0.84 μM for Basal, and 0.88 μM for Max (Figure 2C). Cell counts as determined by Hoechst 33342 fluorescence for each leptochelin A concentration were not affected.

### 3.2. Leptochelin A Inhibits Respiratory Capacity Across Multiple Respirometric Parameters

**Figure 3:**
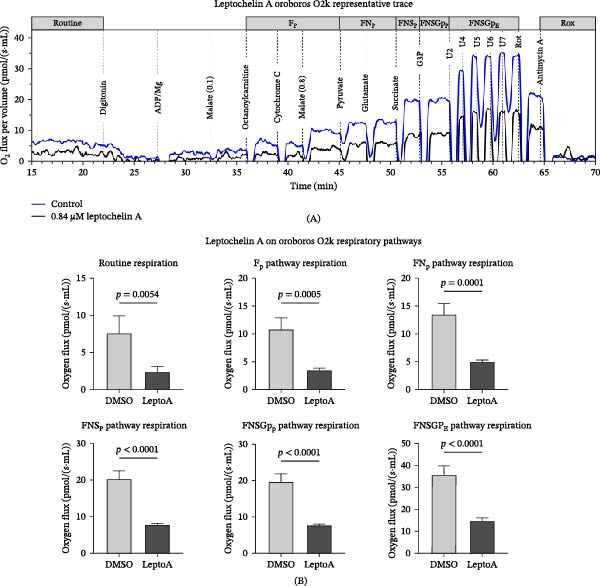
High‐resolution respirometry of fibroblasts treated with leptochelin A. Fibroblasts were treated with 0.84 μM leptochelin A for 24 h and examined with high‐resolution respirometry. (A) Representative trace of a high‐resolution respirometry experiment. (B) Leptochelin A reduces respiratory capacity of dermal fibroblasts across multiple bioenergetic pathways. ADP, adenosine diphosphate; DMSO, dimethyl sulfoxide; G3P, glycerol‐3‐phosphate.

To validate leptochelin A’s impact on mitochondrial respiration and explore its impact on specific respiratory pathways, we utilized high‐resolution respirometry using the same primary fibroblasts as we used in the primary screen. We found that 24 h treatment with 0.84 μM leptochelin A significantly reduced respiratory capacity across multiple bioenergetic pathways, shown in Figure 3.

### 3.3. Leptochelin A Increases Glycolytic Activity

**Figure 4:**
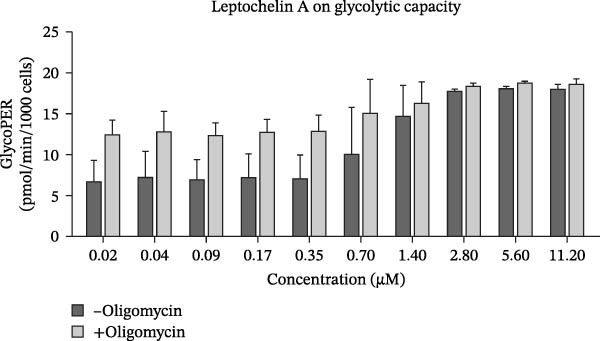
Glycolytic capacity of fibroblasts treated with leptochelin A. Exposure to higher concentrations of leptochelin A for 24 h increases glycolytic capacity in fibroblasts (*n* = 2). GlycoPER, glycolytic proton efflux rate.

Since glycolysis and mitochondrial respiration are often linked, we also examined leptochelin A’s effect on glycolytic activity using the Agilent Seahorse XFe96, as shown in Figure 4. Leptochelin A increases glycolytic activity in a dose‐dependent manner that mirrors its mitochondrial inhibition. Leptochelin A also maximizes the cells’ glycolytic capacity, demonstrated by the lack of difference between basal and oligomycin‐stimulated glycolysis at higher leptochelin A concentrations.

### 3.4. Leptochelin A Increases Mitochondrial ATP and Reduces Membrane Potential and Has No Effect on Mitochondrial Mass and Superoxide Level

**Figure 5:**
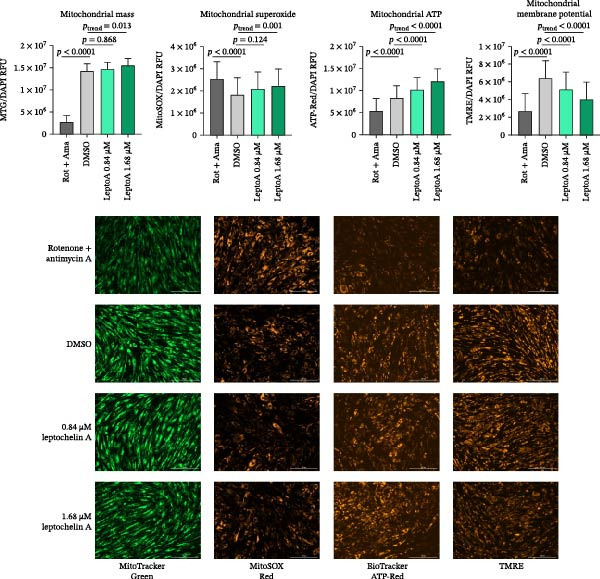
Fluorescent markers of mitochondrial function in fibroblasts treated with leptochelin A. Fluorescent probes allowed relative measurements of mitochondrial mass, superoxide levels, ATP levels, and membrane potential. All values are normalized to cell count (*n* = 3). GlycoPER, glycolytic proton efflux rate.

Fluorescence staining and visualization allow the measurement of unique features of mitochondrial function that are complementary to respirometric outcomes. We used a series of fluorescent probes to determine the effect of 24‐h leptochelin A exposure at 0.84 and 1.68 μM on mitochondrial mass, superoxide level, ATP level, and relative membrane potential. As depicted in Figure 5, we found that 0.84 μM leptochelin A has no effect on mitochondrial mass (*p* = 0.868) or superoxide (*p* = 0.124), although a slight linear trend can be observed across all three concentrations, including the negative control (mitochondrial mass *p*_trend_ = 0.013, mitochondrial ROS *p*_trend_ = 0.001). We also found that leptochelin A increases mitochondrial ATP levels (*p*_trend_ <0.0001) and lowers mitochondrial membrane potential (*p*_trend_ <0.0001) in a dose‐dependent manner.

## 4. Discussion

We utilized a cell‐based phenotypic screening strategy to identify novel modulators of mitochondrial function from a library of marine natural products. Of the candidate hits, we chose leptochelin A for further characterization for its high potency and efficacy. We found that leptochelin A inhibits multiple bioenergetic pathways with an IC_50_ of 0.84 μM. These inhibitory effects are observed without significant generation of reactive oxidative species but are accompanied by an increase in glycolytic activity and mitochondrial ATP and a decrease in mitochondrial membrane potential. To our knowledge, we are the first to report mito‐inhibitory properties of the natural product leptochelin A.

A key objective of this study was the implementation of a phenotypic screening platform using primary human cells as the target for our primary screen. Traditional high‐throughput screens have predominantly relied on biochemical assays (e.g., enzyme activity inhibition and binding affinity measurements) or target‐based approaches using purified proteins. While these methods enable rapid screening of vast compound libraries spanning 100’s of compounds, they often fail to capture the complexity of cellular physiology, leading to hits that may not be effective in a biologically relevant context. In the context of aging biology, which involves multiple biological pathways, this consideration is paramount. Our use of cellular respirometry, often regarded as the gold‐standard measurement of mitochondrial function, as the primary screen ensures a functional output that is widely implicated in the aging process at the cellular level. Indeed, many studies have linked mitochondrial respiration to age‐related conditions and the progression and pathophysiology of age‐related disease (24–26). Alternative cell‐based screening strategies frequently employ immortalized cell lines (e.g., HeLa and HEK293) or non‐human models (e.g., mouse cells and hamster cells), which may exhibit alterations or species‐specific differences that limit translational potential. In contrast, our phenotypic screening approach leverages primary human cells, preserving native cellular environments and aging‐specific pathways. This strategy ensures that identified hits elicit a measurable biological effect in a physiologically meaningful system, significantly improving the likelihood of clinical relevance. Our approach bridges the gap between high‐throughput screening and translational research, offering a more predictive model for drug discovery.

Leptochelin A, recently identified as a structurally novel metallophore from the cyanobacterial genus *Leptothoe*, bears promising therapeutic potential for aging and age‐related conditions. The family of leptochelins A–C exhibits promiscuous metal binding with particular affinity to copper, which is theorized to help the native cyanobacteria survive harsh environments [27]. Metal ions like iron, zinc, copper, and manganese are all important cofactors for metalloenzymes and metalloproteins in the mitochondria (28). Accordingly, metallobiology has garnered increased attention as a promising avenue for the management of the age‐related condition Alzheimer’s disease (AD) [29, 30]. Recent studies have investigated the potential for metal‐chelating agents to slow or halt AD pathology [31]. For example, hydroxypyridinone‐coumarin hybrids have been developed as iron chelators with the ability to restore cognitive function in mouse models of AD [32, 33]. Leptochelin A’s affinity for copper binding coupled with its ability to modulate mitochondrial function makes it a unique compound that should be further evaluated in animal models of aging.

Another notable feature of leptochelin A is the lack of significant toxicity associated with its inhibition of respiratory capacity. Often, the inhibition of respiratory capacity is accompanied by significant damage to mitochondria, either in the form of increased ROS production or overt cytotoxicity. Common inhibitors of respiration, such as the complex‐IV inhibitor sodium azide, are well‐documented to cause severe mitochondrial damage [34–36]. Rotenone and antimycin A, complex‐I and III inhibitors, respectively, are commonly used in experimentation, like in this study, to act as positive controls for significant mitochondrial inhibition and damage [37, 38]. Strikingly, leptochelin A at low concentrations reduced respiratory capacity without triggering ROS production or cellular death. We also observed a concomitant increase in mitochondrial ATP levels, which may be attributable to an increase in glycolytic activity. The uptick in glycolytic activity may be due to compensatory glycolytic upregulation associated with the reduction of oxidative phosphorylation. Nonetheless, from our findings it is evident that leptochelin A inhibits mitochondrial function without overt mitochondrial toxicity. Future studies should examine how prolonged exposure to leptochelin A may impact broader cellular and mitochondrial outcomes. Previous studies demonstrated that prolonged copper depletion may increase ROS production and impair mitochondrial function [39, 40]. Given our findings, it is possible that, at low, nontoxic concentrations, leptochelin A induces mitohormetic responses like increased antioxidant defense systems [39, 41].

The potency for mitochondrial inhibition, low mitochondrial toxicity, and metal‐binding properties of leptochelin A make it a potential candidate as a pharmaceutical agent to target biological aging. We surmise that leptochelin A may be triggering mitohormetic responses, where minor insults to mitochondrial function may confer long‐term benefits rather than detriments—a phenomenon that has been attributed to some interventions that yield positive mitochondrial outcomes. For example, metformin’s ability to inhibit complex‐I is thought to be linked to other mitochondrial benefits such as increased biogenesis and improved morphology [42]. Interestingly, mitohormesis has been specifically linked to improvements in lifespan and healthspan. Work in *C. elegans* demonstrated that minor inhibition of respiratory capacity could extend lifespan [43–45]. Researchers have theorized that the increases in ROS caused by the mitochondrial inhibition enact a protective response with mediators such as SOXO and SIRT1 that are associated with longevity [46, 47]. Others found through targeted depletion studies that mitochondrial inhibition also reduces senescence‐associated phenotypes, including sen‐beta‐gal activity, SASP secretion, and ROS production, which may be another link between mitochondrial inhibition and longevity [48]. Thus, we believe that leptochelin A has the potential to influence biological aging and should be evaluated further in organismal models for its potential ability to improve aging physiology and lifespan.

We acknowledge that this work has its limitations. Dermal fibroblasts, though useful for in vitro investigation of aging and interventions that target aging hallmarks, are less energetic than other cell types and may blunt the apparent impact of mitochondria‐targeting compounds. Other cell types, like skeletal muscle cells or neurons, or disease models of mitochondria‐ or age‐related disease, may have different responses. Though phenotypic screens are useful for downstream translational relevance, they lack insight into target engagement and binding affinity that are often critical in drug development and medicinal chemistry. As such, future studies are required to elucidate the precise molecular mechanism by which leptochelin A inhibits mitochondrial function.

## 5. Conclusion

In conclusion, our study employed phenotypic drug screening leveraging primary human cells to identify compounds that modulate mitochondrial function. Our lead compound, leptochelin A, demonstrated potent inhibitory properties with favorable attributes, including no superoxide generation and increased ATP production. Taken together, the high efficacy and therapeutic potential of leptochelin A highlights the utility of phenotypic screening for the discovery of novel therapeutics to target biological aging. These efforts, along with future studies to evaluate the therapeutic impact and precise mechanism of action of leptochelin A, will advance the development of clinically viable interventions for age‐related decline.

## Acknowledgments

We would like to thank all the staff involved in the attainment and culturing of the SD‐NSC fibroblasts. We would also like to thank all the staff involved in the isolation and curation of the Gerwick Marine Natural Product Library.

## Funding

The work utilizing primary human fibroblasts from the San Diego Nathan Shock Center Clinical Cohort was supported by the National Institutes of Health under Grant P30AG068635. Portions of the work performed by NEA were supported by the National Center for Complementary and Integrative Health of the NIH under Grant F32AT011475.

## Conflicts of Interest

The authors declare no conflicts of interest.

## Supporting Information

Additional supporting information can be found online in the Supporting Information section.

## Supporting information


**Supporting Information** Figure S1: Heatmap of primary screening secondary results. Proton leak respiration (Leak) and ATP‐linked respiration (ATP‐L) as determined from the primary screen of marine natural products. Red indicates a negative change while green indicates a positive change. Table S1: Marine natural products with inhibitory effects on mitochondrial function. Twenty‐five compounds from the primary screen demonstrated at least 20% change in basal or maximal respiration at 10 ug/mL. Compounds listed in this table are ordered by largest change in basal respiration.

## Data Availability

The data that support the findings of this study are available from the corresponding author upon reasonable request.
