# Supplementary material for: Respirometry‐Based Screening of Marine Natural Products Identifies Leptochelin A as a Novel Modulator of Mitochondrial Function
Source: Oxid Med Cell Longev. 2026 May 26;2026:9524037. doi: 10.1155/omcl/9524037 (PMC13213193; doi:10.1155/omcl/9524037)
Supplement: Supplementary file 1 — Supporting Information Figure S1: Heatmap of primary screening secondary results. Proton leak respiration (Leak) and ATP‐linked respiration (ATP‐L) as determined from the primary screen of marine natural products. Red indicates a negative change while green indicates a positive change. Table S1: Marine natural products with inhibitory effects on mitochondrial function. Twenty‐five compounds from the primary screen demonstrated at least 20% change in basal or maximal respiration at 10 ug/mL. Compounds listed in this table are ordered by largest change in basal respiration. [file OMCL-2026-9524037-s001.docx]

**Supplementary Figure 1: Heatmap of primary screening secondary results.** Proton leak respiration (Leak) and ATP-linked respiration (ATP-L) as determined from the primary screen of marine natural products. Red indicates a negative change while green indicates a positive change.

*Notes: ATP, adenosine triphosphate*


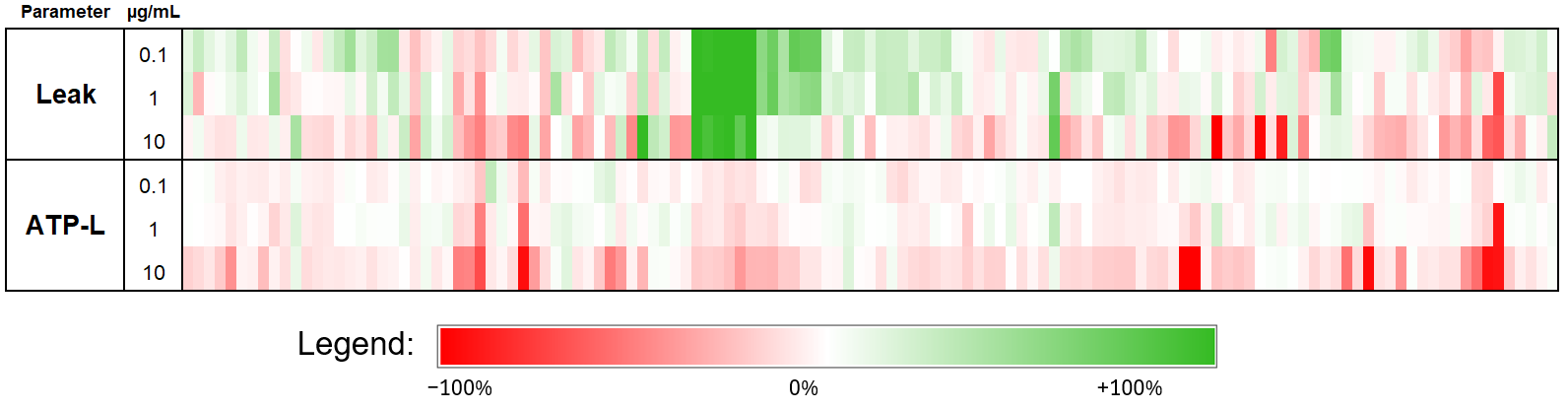


**Supplementary Table 1: Marine natural products with inhibitory effects on mitochondrial function.** Twenty-five compounds from the primary screen demonstrated at least 20% change in basal or maximal respiration at 10 ug/mL. Compounds listed in this table are ordered by largest change in basal respiration.

| **Compound Name** | **%ΔBasal**  **(at 10ug/mL)** | **%ΔMax**  **(at 10ug/mL)** |
| --- | --- | --- |
| Kalkipyrone B | -91.2 | -90 |
| Leptochelin A | -90.3 | -88 |
| Kalkitoxin thioamide alcohol | -89.2 | -94.1 |
| (R,R)-Tanikolide dimer (natural) | -76.8 | -63.9 |
| Hormothamnione diacetate | -67.8 | -47.9 |
| (R,S)-Tanikolide dimer | -64.5 | -48.8 |
| Tasiamide D | -53.8 | -29.4 |
| Kalkipyrone A | -52.8 | -37.3 |
| Microcolin B | -49.2 | -24.8 |
| Hormothamnione triacetate | -47.0 | -31.2 |
| (-)-Malyngolide | -46.6 | -36 |
| Lyngbyabellin N | -41.4 | -22.4 |
| Glycospongosine | -41.3 | -28.7 |
| Psammaplin A | -39.1 | -19.6 |
| R-4-benzyl-3-isobutyryl-5,5-dimethyloxazolidin-2-one | -35.6 | -28.7 |
| Malyngamide I | -33.7 | +1.86 |
| Antillatoxin B (red color) | -32.5 | -42.8 |
| Carmaphycin B enone analogue | -31.9 | -31.7 |
| S-4-benzyl-3-((S)-3-hydroxy-2,2-dimethyloctanoyl)-5,5-dimethyloxazolidin-2-one | -26.4 | -25.3 |
| R-4-benzyl-3-((R)-3-hydroxy-2,2-dimethyloctanoyl)oxazolidin-2-one | -25.3 | -24 |
| Honaucin A | -24.4 | -21.6 |
| Nleu-Val-hexanoate | -23.7 | -19.8 |
| S-4-benzyl-3-((S)-3-hydroxy-2,2-dimethyloctanoyl)oxazolidin-2-one | -23.4 | -16.7 |
| Antillatoxin A | -20.4 | -42.8 |
| Denticulatin B | -20.1 | -18.2 |
